# Cashew Gum: A Review of Brazilian Patents and Pharmaceutical Applications with a Special Focus on Nanoparticles

**DOI:** 10.3390/mi13071137

**Published:** 2022-07-18

**Authors:** Ricardo G. Amaral, Lucas R. Melo de Andrade, Luciana N. Andrade, Kahynna C. Loureiro, Eliana B. Souto, Patrícia Severino

**Affiliations:** 1 Department of Physiology, Federal University of Sergipe, São Cristóvão, Sergipe 49100-000, Brazil; ricardoamaral23@hotmail.com; 2 Laboratory of Pharmaceutical Technology, Federal University of Mato Grosso do Sul, Campo Grande, Mato Grosso do Sul 79070-900, Brazil; rannier.andrade@outlook.com; 3 Department of Medicine, Federal University of Sergipe, Lagarto, Sergipe 49400-000, Brazil; luciana.nalone@hotmail.com; 4 Institute of Technology and Research, University of Tiradentes, Aracaju, Sergipe 49032-490, Brazil; kahynna@live.com; 5 Department of Pharmaceutical Technology, Faculty of Pharmacy, University of Porto, 4050-313 Porto, Portugal; 6 REQUIMTE/UCIBIO, Faculty of Pharmacy, University of Porto, 4050-313 Porto, Portugal

**Keywords:** cashew gum, *Anacardium occidentale* L., polysaccharide nanoparticles, patents

## Abstract

Natural polysaccharides are structures composed of highly diversified biological macromolecules whose properties have been exploited by a diversity of industries. Until 2018, the polysaccharides market raised more than US $ 12 billion worldwide, while an annual growth forecast of 4.8% is expected by 2026. The food industry is largely responsible for the consumption of this plant-source material, produced by microbiological fermentation. Among the used polysaccharides, gums are hydrocolloids obtained from a variety of sources and in different forms, being composed of salts of calcium, potassium, magnesium and sugar monomers. Their non-toxicity, hydrophilicity, viscosity, biodegradability, biocompatibility and sustainable production are among their main advantages. Although Brazil is amongst the largest producers of cashew gum, reaching 50 tons per year, the polysaccharide is not being used to its full potential, in particular, with regard to its uses in pharmaceuticals. Cashew gum (CG), obtained from *Anacardium occidentale* L., caught the attention of the industry only in 1970; in 1990, its production started to grow. Within the Brazilian academy, the groups from the Federal University of Ceará and Piauí are devoting the most efforts to the study of cashew gum, with a total of 31 articles already published. The number of patents in the country for innovations containing cashew tree gum has reached 14, including the technological process for the purification of cashew tree gum, comparison of physical and chemical methods for physicochemical characterizations, and optimum purification methodology. This scenario opens a range of opportunities for the use of cashew gum, mainly in the development of new pharmaceutical products, with a special interest in nanoparticles.

## 1. Introduction

Natural polysaccharides and their derivatives represent a group of polymers characterized by structurally diverse biological macromolecules, which are widely used by different industries due to their versatile physicochemical properties [1,2]. Among the polysaccharides of natural origin, cellulose is the most abundant polymer, representing about 1.5 × 1012 tons (metric ton) of annual biomass [3]. In addition to cellulose, other natural macromolecules (polysaccharides, proteins), such as chitosan, alginate, pectin, starch, collagen and gums, are also of great interest to the pharmaceutical and food industries [2,4,5].

Among natural polysaccharides, natural gums stand out as promising biodegradable polymeric materials, found most often in the woody elements of plants or inside the seed coatings [1,4]. These biomaterials can be used as gelling, thickening, emulsifying and stabilizing agents. Such properties are attributed to their ability to bind to water, to form films and gels, and to encapsulate different compounds, such as flavors, aromas and nutraceuticals [5]. They can also be chemically modified in order to obtain materials useful for the production of drug delivery systems. Other factors, such as low toxicity risk, wide availability in nature and low cost compared to synthetic materials, make natural gum products of great industrial interest [6].

Plant-derived gums are available worldwide [6]. Within this market, the Northeast region of Brazil stands out for its high production rates of cashew gum (95% of national production). The states of the Brazilian Northeast, known as Piauí, Ceará and Rio Grande do Norte, are the holders of the largest *Anacardium occidentale* L. crops, popularly known as cashew tree. This region already has a well-consolidated market for cashew fruit and pseudofruit, with a potential production of around 50,000 tons/year [7].

Cashew gum is, however, a hydrophilic polysaccharide; thus, to be used in pharmaceutical and food products, it needs to be chemically modified or mixed with other polymers. In this review, we discuss the main uses of polysaccharides for pharmaceutical applications, with a special focus on cashew gum, and present the state of the art of patents filled in Brazil that describe the use of cashew gum in pharmaceutical and food formulations.

## 2. Natural Polymers

Polymer is by definition a compound of high molecular weight, resulting from the covalent bond between several hundreds of monomers (monosaccharides) [2]. These monosaccharides are linked by glycosidic bonds and are one of the key biomacromolecules involved in the growth of living organisms, cell–cell communication, cell adhesion and molecular recognition in the immune system [8]. They can also be called glycans, and they differ in the types of constituent monomers, the length of the chains, the type of bonds between the units and the degree of branching [9,10].

**Figure 1:**
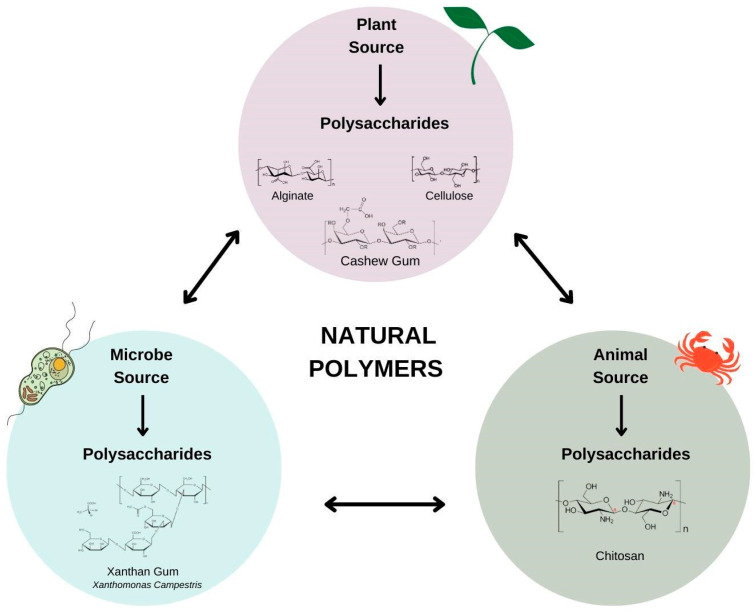
Natural polysaccharides based on sources.

Natural polysaccharides (Figure 1) are produced naturally without human intervention and are found in plants (algae, plant exudate, seeds, fruits, tubers and cereals), animals (bovine, swine and crustaceans), fungi (*P. ostreatus* and *Agaricus blazei*) and bacteria (*Xanthomonas* ssp., *Leuconostoc* spp and *Sphingomonas elodea*) [7].

Due to their versatility, simple extraction process, biocompatibility and easy biodegradation, natural polysaccharides are of scientific and industrial interest as pharmaceutical excipients for the formulation of cosmetics, conventional-release drugs, modified-release drugs and a diversity of foodstuffs. In addition, they are referred to as biological macromolecules, described in the literature as immunological [11], antioxidant [12], anti-tumor [13], antidiabetic [14], anti-inflammatory [15], antiviral [16], antifungal [17] and anticoagulant [18] agentes, and also show cardiovascular [19] and hepatoprotective [20] effects. 

Currently available sources report that these polysaccharides and oligosaccharides will have an annual growth rate of over 5% between 2020 and 2030, led primarily by the food and beverage industries [21]. In 2018. this market reached US $5.7 billion in Asia, followed by Europe, North America and Latin America markets. However, comparing both products, polysaccharides will dominate the market, with an estimated growth of 4.6% per year. In 2018, they reached a total of US $6.4 billion and have bacteria as their main source of production to supply the world market, followed by those obtained from plants, making up the second largest revenue [22].

There are several types of polysaccharides (Table 1) with potential pharmaceutical uses, such as Alginate, Agaranas, Carrageenans, Tragacante, Guar, Carob, Tamarind, Pectins, Starch, Inulin, Hyaluronic acid, Heparin, Chitin, Chitosan, Glucans, Xanthan, Dextran, Gelana and Gum. Among these, the most frequently used in the industry are mucilages, chitosan with different degrees of acetylation, alginate, pectin, starch, and different types of gums [2,23,24].

### 2.1. Gums

Gums are natural polysaccharides that are classified according to their charge (ionic or non-ionic), shape (branch or short branch), origin (seeds, plant exudate, microbial or algae), gelling behavior (cold, heating or reversible) and chemical structure (galactomannan, glucomannan, uronic acid, monoglycans or heteroglycans) [22].

Also known as gum hydrocolloids, they are a product developed by the plant as a defense mechanism in response lesions occurring mainly on the stem or due to unfavorable conditions such as the drying of the cell walls, as a form of pathological gummosis [4]. This biomaterial is composed, in general, of calcium, potassium and magnesium salts, and sugar monomers such as galactose and arabinose, from hemicellulose [24,49]. Xanthan, agar, Arabic, carob, ghatti, tragacanth, karaya, carrageenan and guar gums are amongst the most commonly occurring gums on the market [30].

Several natural gums are used by the food and pharmaceutical industries, e.g., as emulsifiers, disintegrants, emollients and additives. Because of their biodegradability, biocompatibility, non-toxicity, hydrophilicity and high viscosity, they are present in many pharmaceutical products as a component for suppositories (agar), in cosmetic products (gum Arabic, xanthan gum), as a stabilizer in toothpaste (xanthan gum), and in modified drug delivery systems (guar gum, locust bean gum, xanthan gum) [49]. Table 1 summarizes examples of natural gums and their applications in pharmaceutical and food products. Among all the studied and applied gums, some are still little explored in both areas, such as cashew gum.

#### 2.1.1. Cashew Gum

Cashew gum is a complex heteropolysaccharide obtained from *Anacardium occidentale* L., a plant native to Venezuela and Brazil (North and Northeast). It was introduced in Africa and India by the Portuguese, and today it is spread over several Asian regions (Vietnam, United Republic of Tanzania and Indonesia) [50].

The cashew tree is a large tree that can reach up to 20 m in height. Its parts have several therapeutic uses; however, the fruits or cotyledons, known as cashews and cashew nuts, are the main economically valuable product; they are edible and are used as a snack or in the manufacture of sweets, having a higher competitiveness over the production of nuts, peanuts, hazelnuts and pistachios [51]. After the nut, the second main product of the cashew tree is the cashew nut shell liquid (CNSL), followed by the juice from the pulp of the pseudofruit or pendulum, called the cashew [50,51].

While these products are of unquestionable economic value, the cashew bark also has high commercial potential from the extraction of its exudate by incision, producing a yellow or reddish-brown gum resin. Exuded gums are polysaccharides produced by plant epithelial cells when the cortex is physically injured or suffers any microbial attack. The cashew gum is thus produced by the epithelial cells of the cashew tree in response to mechanical stimuli or pathogens [52].

Some studies report the possibility of using cashew gum in several sectors; however, its applications in an industrial environment are not well established. Although cashew gum has been explored since 1970, it is only since the 1990s that it has received increased interest from scientific community, mainly due to structural and chemical similarity with gum Arabic. The gum Arabic, also known as gum Acacia, is considered the oldest and the best known among natural gums; however, it has high cost, and occasionally its import is compromised by the difficulty of supply due to climatic, economic and political problems in the African producing region [53,54].

The cashew gum, in addition to the structural (branching) and chemical (component sugars) similarities with gum Arabic, has an important differential: it has high availability in the Northeast region of the Brazilian territory, which can generate profits in the period between the cashew season [55,56].

**Figure 2:**
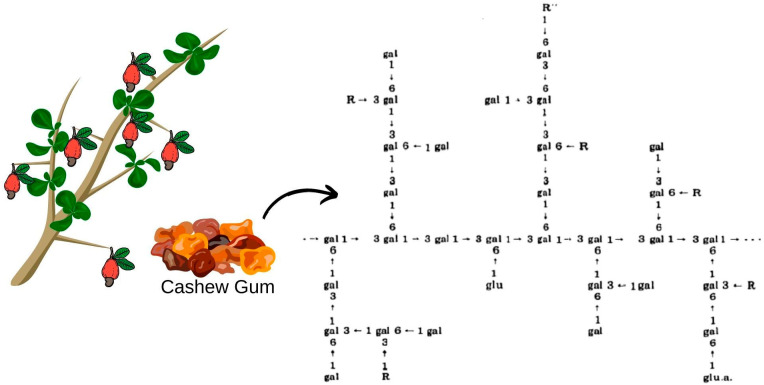
Possible structure of *Anacardium occidentale* gum, L. R represents D-mannose, D-xylose, L-rhamnose, L-arabinose or 1–2 linkage with the arabinose chain. R” represents D-glucose or D-glucuronic acid.

Cashew gum extracted from trees in the northeast region was characterized as a branched heteropolysaccharide containing in its structure (Figure 2): β-D-galactose (72–73%), α-D-glucose (11–14%), arabinose (4–6.5%), rhamnose (3.2–4%) and glucuronic acid (4.7–6.3%) by mass percentage [57]. In addition, it presents advantages in relation to gum Arabic, such as: lower ash content, indicating a lower degree of impurities even in the non-purified sample; higher protein content, which can provide better emulsifying properties, desired in the encapsulation processes of oils and aromas; high fiber content, being higher than gum Arabic and other traditional materials; and lower viscosity after extrusion, which can contribute to better dispersion and solubility in solutions [58,59]. Therefore, cashew gum could be indicated as a substitute for gum Arabic, and its commercial standardization would raise Brazil to a significant exporter in the international market, thus reducing the import dependency and creating a highly competitive product in its export share [58].

**Figure 3:**
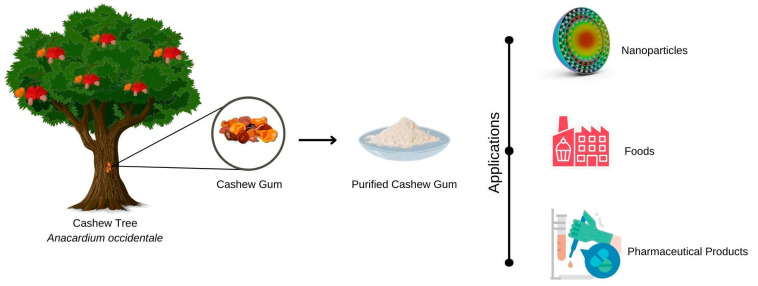
Representation of potential applications of cashew tree gum in different fields, such as food, pharmaceutical excipients, and micro- and nanostructured systems.

Cashew gum can be used, for example, in the manufacture of (Figure 3) pharmaceutical products, foodstuffs, cosmetics, adhesives and, more recently, in nanotechnology [57]. 

**Figure 4:**
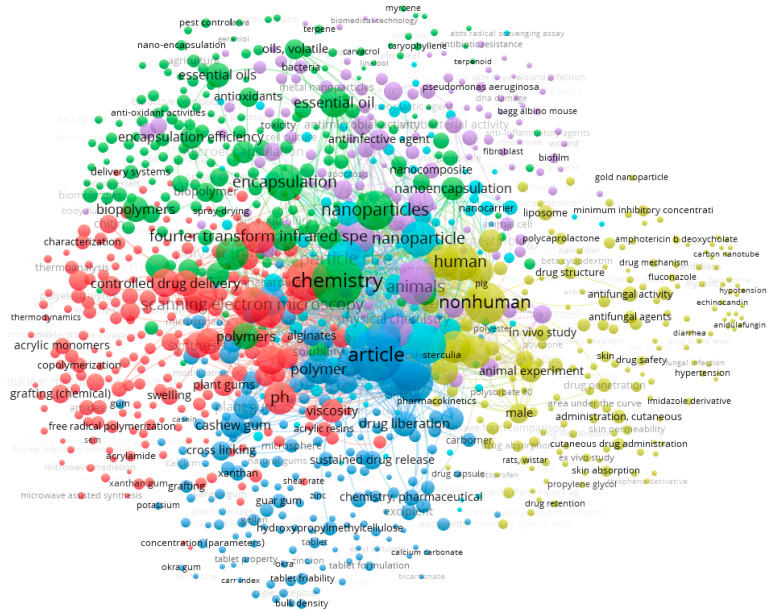
Bibliometric map obtained by VOSviewer software version 1.6.16 (https://www.vosviewer.com), using “cashew gum” AND “pharmaceutics” as keywords, recorded from Scopus database.

A survey in Scopus database generated a total of 824 published papers that combine both “cashew gum” AND “pharmaceutics”. VOSviewer was used to obtain the bibliometric map shown in Figure 4 [60,61]. Six clusters of terms were generated, demonstrating the wide range of uses of this polysaccharide in biomedical applications (e.g., development of nanocomposites, biodegradable films responsive to stimulus materials, gel dressings and controlled release tablets, and chemical engineering of new polymers, oxidation, sulfation, carboxymethylation, acetylation or copolymerization).

#### 2.1.2. Applications of Cashew Gum in the Development of Micro- and Nanoparticles

**Figure 5:**
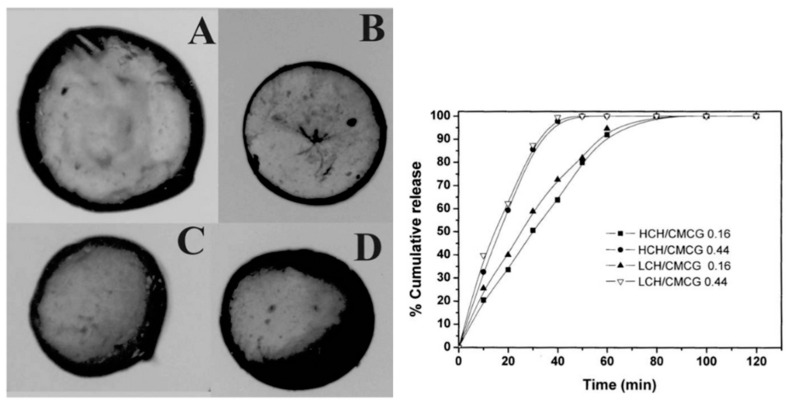
Optical microscopy of different chitosan/carboxymethyl cashew gum (CH/CMCG) microspheres. (**A**) HCH/CMCG 0.16; (**B**) HCH/CMCG 0.44; (**C**) LCH/CMCG 0.16; and (**D**) HCH/CMCG 0.44 and effect of chitosan (CH) molar mass and degrees of substitution (DS) of chitosan/carboxymethyl cashew gum (CMCG) on in vitro release profile of BSA (Adapted from Magalhães et al. (2009) [63], Copyright© 2009, Elsevier Ltd).

Cashew gum (CG) has been recently suggested as a biopolymer for the production of nanoparticles (NPs) and nanogels through various methods, with nanoprecipitation the most frequently used. In addition to several advantages, such as low cost, biocompatibility and self-assembly, several studies have highlighted the gastroprotective, anti-inflammatory and healing properties of cashew gum (Table 2) [62]. Magalhães et al. (2009) [63] developed chitosan/carboxymethyl cashew gum (CH/CMCG) microspheres (Figure 5) with different degrees of substitution and evaluated the release rate of the drug incorporated into the CG/chitosan matrix, which revealed high release rates of microparticles prepared using high density carboxymethyl CG, while those prepared with a low density (DS = 0.16) promoted a prolonged release of the drug.

De Oliveira et al. (2020) [64] combined alginate and cashew gum for the production of nanoparticles by spray-drying for the loading of *Lippia sidoides* essential oil. The obtained particles were in the range of 223–399 nm, and the encapsulation efficiency reached 55%, achieving 95% of the oil released within 30–50 h in vitro.

In recent years, the use of coacervation has become one of the most effective methods for micro/nanoencapsulation in pharmaceutical and food industries. This process is formed by a three-phase system in relation to the solvent, the active and the coating material, which involves four steps: (i) preparation of aqueous solution containing two or more polymers, (ii) mixing the hydrophobic phase with the aqueous solution of the polymer, (iii) changing the pH and temperature to induction of coacervation and phase separation and (iv) resistance of polymer matrices (temperature, crosslinking or dissolving agent) [65].

Gomez et al. (2016) [66] used the coacervation method for the development of CG and gelatin microcapsules for the encapsulation of shrimp lipid extract (astaxanthin). The study described the production of microparticles of approximately 32.7 ± 9.7 µm and an astaxanthin encapsulation efficiency of 59.9 ± 0.01%. The results showed that the presence of cashew tree gum in the microparticles resulted in the delay of the lipid extract degradation compared to non-encapsulated ones, thus demonstrating a high potential for the use of cashew gum to replace gum Arabic in the development of new technological products as a food ingredient.

Araruna et al. (2020) [67] developed silver nanoparticles combined with cashew gum (CG) and carboxymethylated cashew gum (CCG), exploring the role of microwave heating in the process of reducing silver ions at different pH (10 and 11) and the activity of the gum in stabilizing the nanoparticles. The cashew gum carboxylation process was carried out by back titration to GC mixed in 10 M NaOH, followed by the addition of monochloroacetic acid and by neutralization with 1M HCl. The synthesis of the nanoparticles was performed by mixing GC and GCC with an AgNO3 solution (1 mM) in a domestic microwave oven with exposure to microwaves at 2450 MHz for 3 min, which led to the reduction of Ag+ ions and the formation of nanoparticles: CGAgNP and CCGAgNP.

**Table 2:** Applications of cashew gum (CG) for the loading of bioactives in the production of micro/nanoparticles.

Variation of CG	Polymers	Encapsulated Bioactive	Method	Size	Objective	References
CG carboxymethylated	Chitosan	Bovine serum albumin	Nanoprecipitation	500–580 μm	Albumin release due to swelling behavior.	[63]
CG copolymerized	Acrylic acid	-	Self-embedding copolymerization	71–603 nm	Prepare CG particles and acrylic acid and evaluate the responsive pH behavior.	[68]
CG	Chitosan	*Lippia menosides* essential oil	Emulsion	1.50–1.56 mm	Larvicidal activity.	[69]
CG	Chitosan	*Lippia menosides* essential oil	Emulsion	219–674 nm	Effects of spray-drying and the concentration of polymers in the preparation of particles.	[70]
CG	Alginate	*Lippia menosides* essential oil	Emulsion	223–399 nm	Effects of spray-drying and the concentration of polymers in the preparation of the particles.	[64]
CG acetylated	-	*Lippia menosides* indomethacin	Self-assembly	140–179 nm	Evaluation of the release profile of the produced particle confirming its application in drug release.	[71]
CG	Inulin	Ginger essential oil	Emulsion	13.43–18.52 μm	To evaluate the influence of CG and inulin, in powder particles, in order to obtain functional products with ginger essential oil.	[72]
CG copolymerized	N-isopropylacrylamide (97%)	-	Radical polymerization	11–23 nm	Copolymerize the cashew gum in order to make it sensitive to stimuli for the purpose of drug administration.	[73]
CG	Type B gelatin	Carotenoid	Emulsion	113 μm 23–42.4 μm	Encapsulate astaxanthin in the polymer particle without the use of solvents.	[66]
CG acetylated	-	Diclofenac diethylamine	Nanoprecipitation/Dialysis	79.32 nm/ 302 nm	Encapsulate the drug using different methodologies and compare them, in order to develop a transdermal delivery device.	[74]
CG	-	Omega 3	Emulsion	29.9 μm	Substitute potential for CG in the encapsulation of Omega 3.	[55]
CG	Maltodextrin	Green tea leaf extracts	Emulsion	2.50–3.64 μm	Develop alternative microcapsules of green tea extract for the food industry for health benefits.	[75]
CG	-	D-limonene	Emulsion	17–26.01 μm	Evaluate the effects of high dynamic pressure (APD) on emulsifying and encapsulating characteristics of CG.	[76]
CG acetylated	Monobasic sodium phosphate, bibasic sodium phosphate and sodium lauryl sulfate	Amphotericin B	Self-assembly	50–900 nm	To investigate the influence of temperature, time and proportion of the acetylating agent on the acetylation of cashew gum as well as the influence of the degree of substitution of derivatives on their properties.	[77]
CG	Poly (L-lactide)	Amphotericin B	Nanoprecipitation and Pickering Emulsion	100–3500 nm	Combine different particle production methodologies to encapsulate amphotericin B and improve its oral absorption, enhancing the treatment of leishmaniasis.	[78]
CG acetylated	-	Epi-isopiloturine	Dialysis	107–156 nm	Increase the solubility of the alkaloid and enable its controlled release.	[79]
CG acetylated	-	Indomethacin	Pickering emulsion	263.7–325 nm	Evaluate the points that make it possible to develop CG particles acetylated by Pickering Emulsion without surfactant, with and without Indomethacin.	[80]
CG	Gelatin	Green coffee oil	Complex coacervation	13.9–25.7 μm	Produce green coffee oil microcapsules by complex coacervation for addition to juices.	[81]
CG copolymerized	L-Lactide	Amphotericin B	Dialysis	223–233 nm	Produce copolymerized CG particles by dialysis to encapsulate amphotericin B and compare with previous study.	[82]
CG	Potassium hexacyanoferrate (II) trihydrate and iron (III) chloride	-	Nanoprecipitation	63.5–85.0 nm	Develop a hybrid nanomaterial (Prussian Blue + CG (used to stabilize the matrix)) to act as an electrochemical sensor for the oxidation of some drugs.	[83]
CG Carboxymethylated	Cashew gum and carboxymethylated	cashew gum	Green synthesis	100.9–144.7 nm	Antibacterial activity of silver nanoparticles based on cashew gum and carboxymethylated cashew gum.	[67]

Natural polymers have also been intensively evaluated in the development of micro- and nanoparticles for pharmaceutical purposes [84,85,86,87]. Nanoparticles are colloidal systems with diameters of 1–1000 nm that show important advantages in the improvement of the bioavailability of loaded drugs while also reducing the risk of toxicological events, provided that a lower drug dose will be required to reach the site of action given that nanoparticles have site-specific delivery features [88]. Nanoparticles obtained from naturally occurring polymers are also reported to be stable and useful in the protection of bioactives against degradation [86]. Polysaccharides are among the most used materials in the development of nanoparticles due to their biocompatibility, biodegradability and structural flexibility [89]. A study performed by Richter et al. (2020) [82] used different ratios of copolymers of cashew tree gum and L-lactide to carry amphotericin B (AmB) by the self-assembled method. The nanoparticles showed mean size values between 230 and 250 nm, with the capacity to inhibit all tested strains of *C. albicans*. Pickering emulsion was used to produce cashew gum-poly-l-lactide (CGPLAP) nanoparticles copolymer by Richter et al. (2018) [78] for the loading of amphotericin B (AmB) and was compared to a commercial AmB formulation. The CGPLAP nanoparticles resulted in an encapsulation efficiency of 21% and 47% using the initial AmB concentrations of 5 and 10 mg (respectively). These nanoparticles were stable for up to 28 days under refrigeration.

**Figure 6:**
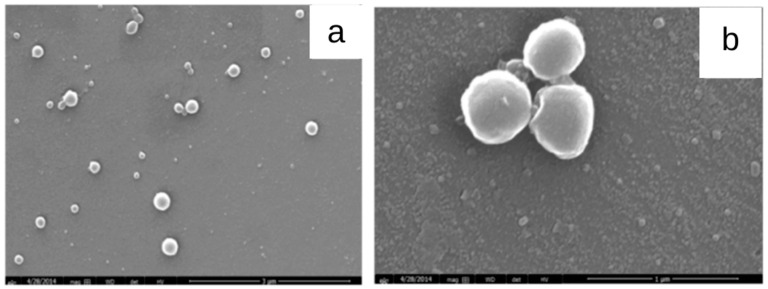
SEM images of acetylated cashew gum (ACG) nanoparticles (**a**) without and (**b**) with diclofenac diethylamine (DDA) (adapted from Dias et al. (2016) [74], Copyright© 2009, Elsevier Ltd).

Dias et al. (2016) [74] developed cashew gum nanoparticles (Figure 6) using the nanoprecipitation method followed by dialysis, with the aim to encapsulate diclofenac diethylamine. An encapsulation efficiency greater than 60% was obtained. The loaded nanoparticles of particle sizes between 23 nm and 1.5 mm showed biocompatibility when evaluated in OSCC cell lines, demonstrating that cashew gum may be a promising polysaccharide to develop delivery systems for anti-inflammatory drugs.

Pickering emulsions were also used by Lima Cardial et al. (2019) [80] in the development of nanoparticles containing acetylated derivatives of cashew gum, with the aim of loading indomethacin. The CG nanoparticles carrying the drug showed particle size and zeta potential influenced by the different degrees of acetylation of the polysaccharide, presenting an encapsulation efficiency of about 50% for indomethacin.

From these studies, there is still a range of opportunities to be explored using CG in nano- and microemulsion systems. Cashew gum as a potential substitute for other gums, added to the large growing market in production, mainly in the Brazilian northeast, make the development of new products a promising field of pharmaceutical research using this polysaccharide.

## 3. Cashew Gum Patent Perspectives in Brazil

Cashew gum (CG) has been tested for various applications in a range of industries (plastics/polymerics, agri-food and pharmaceuticals). The use of this biopolymer in scientific research has become an object of interest mainly in the last decades due to its promising results as a food additive and in the development of nanoparticles for drug encapsulation. Different techniques to obtain, modify and use cashew gum have been developed and patented. The patents related to the use of cashew gum and its applications are presented in Table 3.

The study published on the website of the National Institute of Industrial Property (INPI) showed a total of 14 patents between 1990 and 2018 that relate isolation method, technological development and applications of cashew gum. The first patent related to cashew tree gum was patented by Paula and Rodrigues in 1990 [52]. The inventors disclosed the method of isolating cashew gum from the raw gum exuded from the tree. By isolating the polysaccharide composing the gum, it was separated from impurities by applying steps of dialysis, lyophilization and pH adjustment. Mothé (2000) [102] disclosed the invention of the process of obtaining and the composition of the purified cashew tree gum. The purification process results from solubilization, centrifugation and addition of ethyl alcohol to precipitate the polysaccharide and, finally, vacuum drying to obtain the purified gum, which appears as a white powder that is odorless and has a mild taste. An invention related to a superabsorbent hydrogel was disclosed by Rubira et al. (2004) [100]; the patent describes the chemical modification of cashew gum with glycidyl methacrylate, leading to the insertion of carbon–carbon unsaturation in the cashew gum structure. The development of a hydrogel matrix associating cashew gum copolymerized with acrylamide showed a superabsorbent material and delayed water loss. Soares et al. (2014) [104] disclosed the invention of a cashew gum and chitosan hydrogel without modification in the chemical structures of the polysaccharides. The cashew gum and chitosan hydrogels showed a water absorption capacity of about 270% (1:4) and 320% (2:3) of the initial weight under immersion in water for 24 h, showing the practical use of this material in the pharmaceutical industry for the development of new materials for application in topical dressings.

Sobrinho et al. (2015) [97] patented an invention of a new excipient containing a processed blend of cashew tree gum and chitosan for the development of topically applied products. The mucoadhesive polymer blend described by the inventor was obtained by solubilizing cashew tree gum in distilled water and chitosan in acetic acid (2%) followed by rotational evaporation and lyophilization steps. Subsequently, the mucoadhesive and release properties were evaluated by incorporating the excipient into a pharmaceutical form for the development of a mucoadhesive product containing pilocarpine; the results showed that the mucoadhesive formulation containing GC/chitosan and pilocarpine had a mucoadhesion time of 510 min. According to the invention, the polymeric blend (cashew gum and chitosan) had high performance, high safety and low cost, granting it mucoadhesive and modified release properties.

The use of cashew gum in the encapsulation of bioactives has been patented in the last decades. Torres et al. (2016) [96] invented cashew gum microcapsules encapsulating green tea extract (*Camelliasinesis*), with the aim of protecting the phytochemicals of the tea extract. In addition to the preservation of bioactive compounds, the encapsulation process opens new markets for the use of cashew gum, which is of low cost and easily accessible.

In addition to micro- and nanoparticles, some therapeutic/nutraceutical properties of cashew tree gum have been demonstrated in studies. A patent disclosed by Mothé et al. (2016) [57] used cashew gum as a food additive in chocolate production. This invention used cashew gum as a partial substitute for cocoa butter normally used for the production of chocolates, creating a chocolate with fewer calories and with potential nutraceutical effects on human health. The use of cashew gum has also been reported in the development of biomaterials with potential use in surgical applications and as a support (scaffold) for cell growth in the process of tissue regeneration. The scaffold was developed using a cashew gum solution modified with phthalic anhydride (2.5–4.0%) and mixed with a chitosan solution dissolved in acetic acid (2.5%). The in vitro cell culture showed that the scaffold with cashew gum is an effective support for the growth of mesenchymal stem cells (MSC). Positive results in the cytotoxicity test indicated biocompatibility and non-toxicity, making cashew gum a possible component of synthetic matrices for therapeutic purposes and tissue repair.

Silvia et al. (2018) [90] published an invention from purified cashew gum for the preparation of nanotechnology-based products. The patent disclosed the extraction of cashew tree gum followed by the process of purification and chemical modification through the chemical insertion of acetyl groups transforming the hydrophobic cashew gum.

## 4. Conclusions

A review of recent works, patents and the main techniques for the development of micro- and nanoparticles using cashew gum was presented, highlighting the potential of this biopolymer in the creation of new products applied to human health. This review emphasizes that exploitation of cashew gum has the potential to widen the food and pharmaceutical markets, especially the domestic Brazilian markets. A number of patents covering isolation methods, composition, technological developments and applications of cashew gum have been filled in the last decades, with the potential to create a portfolio of products of high market value.

## Figures and Tables

**Table 1:** Pharmaceutical and food applications of the main gums on the market.

Name of Gum	Pharmaceutical Applications	Food Applications	References
Agar	Compound for suppository, suspension and emulsification, disintegrant, lubricant and laxative.	Dairy, meat and confectionery products.	[25,26]
Arabic	Suspending agent, emulsifying agent, binder in tablets, emollient in cosmetics, osmotic drug delivery.	Chocolate, beverages and soft drinks.	[27,28,29]
Carrageenan	Gelling agent, stabilizer in emulsions and suspensions, toothpaste, demulcent and laxative.	Ice cream, milk shake mixes, cream cheese, dairy desserts and chocolate milks.	[30,31,32]
Ghatti	Binder, emulsifier, suspending agent.	Dressings, processed cheese and beverages.	[33,34]
Guar	Binder, disintegrant, thickening agent, emulsifier, laxative, sustained release agent, colon-targeted drug delivery, cross-linked microspheres.	Drinks, sauces, soups, ketchups and mayonnaises.	[35,36,37,38]
Karaya	Suspending agent, emulsifying agent, dental adhesive, sustaining agent in tablets, bulk laxative, mucoadhesive.	Cheese spreads, and as a binder for low-calorie, dough-based products such as pasta and bread.	[39,40,41]
Locust bean	Thickener, stabilizer and controlled release agent, formulation of oral delivery systems based on tablets, hydrogels and multiparticulate systems.	Ice cream, bakery products, edible films/coating, hot-prepared sauces, soups, dressings, ketchups and mayonnaise.	[33,42,43]
Tragacanth	Suspending agent, emulsifying agent, demulcent, emollient in cosmetics and sustained release agent.	Salad dressings, bakery emulsions, fruit beverages and sauces.	[44,45]
Xanthan	Suspending agent, emulsifier, stabilizer in toothpaste, ointments, sustained release agent, buccal drug delivery system.	Ice creams, pasteurized process cheese dips, frozen desserts and beverages.	[46,47,48]

**Table 3:** Patents filed in Brazil that contain ‘gum AND cashew’ in the title and summary.

Inventor	Request	Deposit	Title	Reference
Federal University of Pernambuco	BR 10 2018 014996 2	23/07/2018	Micro and nanoparticles of acetyled cashew gum biopolymer for pharmaceutical delivery.	[90]
Goiás Federal University	BR 10 2017 020813 3	28/09/2017	Biodegradable plastic based on cashew gum for application as packaging for dehydrated commercial products.	[91]
Federal University of Piauí	BR 10 2017 012139 9	08/06/2017	Porous matrix developed based on chitosan and polysaccharide exudate from *Anacardium occidentale* L. modified with phthalic anhydride for cultivation of mesenchymal stem cells.	[92]
Goiás Federal University	BR 10 2017 007322	10/04/2017	Water-soluble nanoporous solid foam for controlled release of drugs into mucous membranes.	[93]
Cheila Gonçalves Mothé	BR 10 2016 027801 5	25/11/2016	Chocolate food compositions containing cashew gum, in bars, bonbons and powdered chocolate, useful as functional and nutraceutical food.	[94]
Federal University of Ceará	BR 10 2016 018308 1	09/08/2016	Nanoencapsulated waste from the fruit processing industry in a polyelectrolytic matrix of cashew gum and chitosan for use as a coating on minimally processed fruits.	[95]
Federal University of Ceará	BR 10 2016 002436 6	03/02/2016	Encapsulation of green tea (*Camellia Sinensis*) by “spray dryer” with cashew gum / maltodextrin.	[96]
Federal University of Pernambuco / Federal University of Piauí	BR 10 2015 027337 1	28/10/2015	Mucoadhesive polymer blend for prolonged drug release.	[97]
Federal University of Rio Grande do Sul	BR 10 2015 005684 2	13/03/2015	Process of obtaining a biodegradable flocculant from cashew gum and its use for water and effluent treatment.	[98]
Federal University of Pernambuco / University of São Paulo	BR 10 2014 014009 3	10/06/2014	Hydrogel based on natural polysaccharides, processes and uses.	[99]
National Council for Scientific and Technological Development	PI 0404265-4	29/09/2004	Superabsorbent hydrogels made from modified cashew gum and acrylamide.	[100]
Mineral Technology Center	PI 0304986-8	15/09/2003	Process for using cashew gum as a depressant in flotation of limestone minerals.	[101]
Cheila Gonçalves Mothé	PI 0004114-9	12/09/2000	Process of obtaining purified cashew gum and composition of purified cashew gum.	[102]
Federal University of Ceará	PI 9005645-0	31/10/1990	Isolation method of cashew gum (*Anacardium occidentale* L.).	[103]

Source: INPI, 2022.

## Data Availability

Not applicable.
